# Implementation of an open adoption research data management system for clinical studies

**DOI:** 10.1186/s13104-017-2566-0

**Published:** 2017-07-06

**Authors:** Jan Müller, Kirsten Ingmar Heiss, Renate Oberhoffer

**Affiliations:** 1 Institute of Preventive Pediatrics, Technische Universität München, Uptown München-Campus D, Georg-Brauchle-Ring 60/62, 80992 Munich, Germany; 2 OpenCampus GmbH, Kastenbauerstraße 2, 81677 Munich, Germany

**Keywords:** Clinical trials, Data management, Open-source software

## Abstract

**Background:**

Research institutions need to manage multiple studies with individual data sets, processing rules and different permissions. So far, there is no standard technology that provides an easy to use environment to create databases and user interfaces for clinical trials or research studies. Therefore various software solutions are being used—from custom software, explicitly designed for a specific study, to cost intensive commercial Clinical Trial Management Systems (CTMS) up to very basic approaches with self-designed Microsoft^®^ databases.

**Findings:**

The technology applied to conduct those studies varies tremendously from study to study, making it difficult to evaluate data across various studies (meta-analysis) and keeping a defined level of quality in database design, data processing, displaying and exporting. Furthermore, the systems being used to collect study data are often operated redundantly to systems used in patient care. As a consequence the data collection in studies is inefficient and data quality may suffer from unsynchronized datasets, non-normalized database scenarios and manually executed data transfers.

**Conclusions:**

With OpenCampus Research we implemented an open adoption software (OAS) solution on an open source basis, which provides a standard environment for state-of-the-art research database management at low cost.

## Background

Data management in clinical trials is a complex process that, apart from the technical point of view, has to deal with many ethical principles and guidelines [1–3]. Especially since clinical trials are conducted globally to reduce health burden [4]. Therefore various software solutions exist, ranging from customized software, explicitly designed for a specific study, to cost intensive commercial Clinical Trial Management Systems (CTMS), down to self-made Microsoft Access/Excel^®^ based approaches. The latter is still the dominant solution in smaller research institutions, yet this cost effective approach lacks the principles of open access, documentation accountability, transparency and the basic data security elements which comprise the cornerstones in research data management [1, 3].

Those directives aim to protect patients and improve research standards [5], however the use of those CTMS is expensive and adaptions to these systems are time consuming. In addition to the bureaucratic endeavor, these obstacles have led to a decline in the number of clinical trials by independent academic groups [5].

Therefore some articles have questioned the use of free Open Source Software like OpenClinica^®^ versus Commercial Systems like Oracle^®^ Clinical that charge for user licenses and maintenance support [6, 7].

Products that offer cost-effective and customizable solutions are rare. Our institution therefore opted to use the OpenCampus research data management system (RDMS) to successfully manage and administrate our clinical trials in accordance with the legal and ethical guidelines of the scientific community [1]. The base technology used for that RDMS is the Open Source software Drupal. The architecture of the open adoption software (OAS) allows the user after a brief instruction to develop their own web based data management system. The architecture and applicability of the OAS is outlined in this article to address the need for database solutions in clinical studies. The examples presented in this article are recently complete or ongoing studies in our Institution established 2014.

## Methods

### Basic architecture

**Fig. 1:**
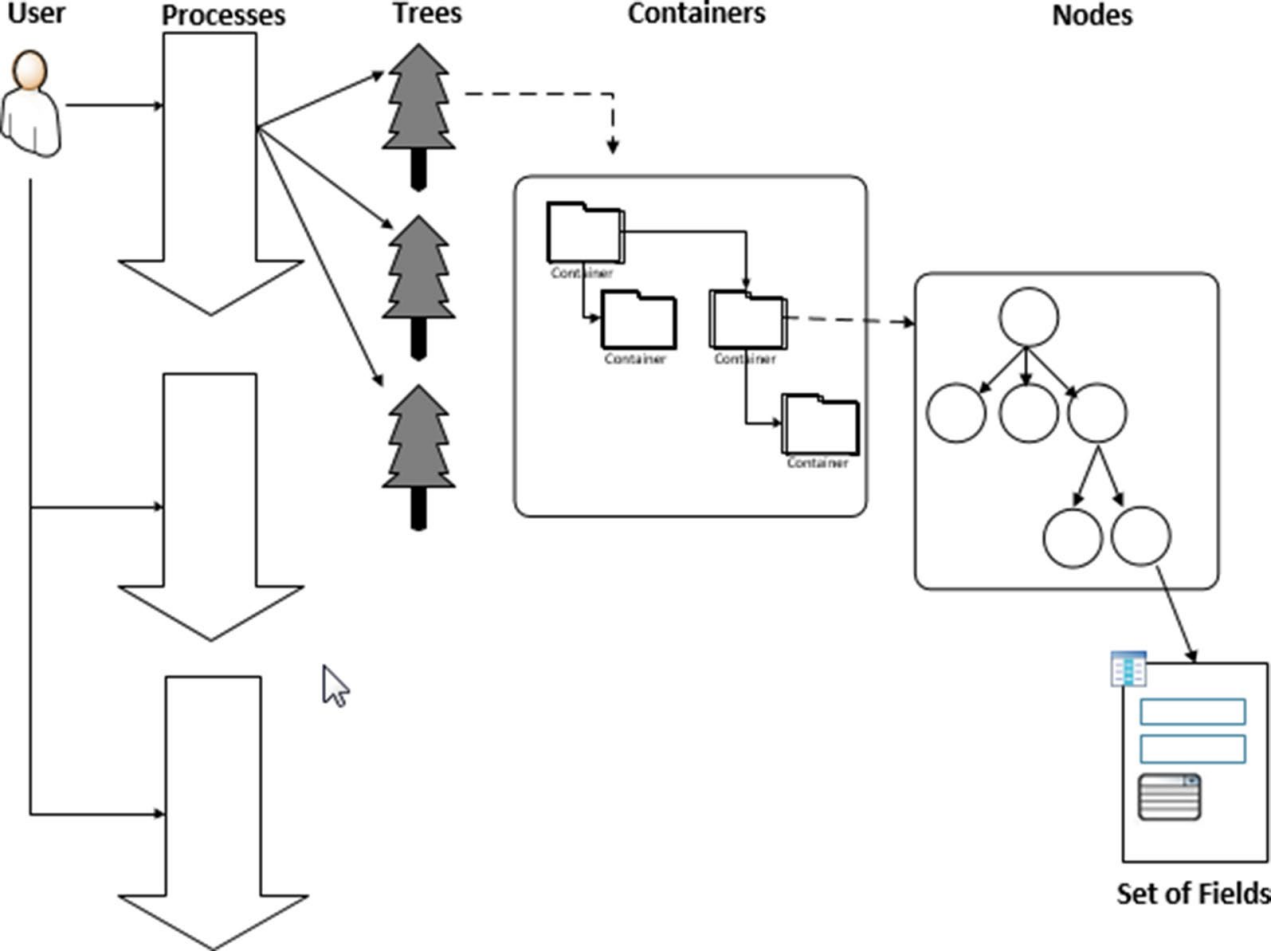
Basic architecture of the research database management system

The configurable elements (tiers) used to develop the RDMS in OpenCampus are basically three types: forms (stored as nodes), Trees and input/output plugins (Fig. 1). A form or node contains fields that allow data entry in a given format (e.g. text field, drop down, reference selector). All forms are stored in the database without being tied to a specific software function or study.

The second tier is the Study Tree, which is a structural definition of containers and their relations to each other. Containers can hold forms, store conditions and actions. This allows to structure the data, build complex rule sets and develop software functions on an “if-this-then-that” basis. This basically describes an “electronic patient record” paired with software information that is needed to execute automatic actions, such as creating a medical report or e-mail notification.

The third tier provides Plug-Ins to use different formats for information input/output and actions. Plug-Ins can read and write the data stored in forms for conversion into email, PDF, mediastream, ECG data, Ultrasound data etc. or simple reformat information (replace text with image). Plug-Ins are also used to manage input/output of data to other systems in this way operating as middleware.

Every research project or clinical trial can always be mapped with the model shown in Fig. 1, using Forms (Nodes), Trees and the respective actions defined through plugins.

As all of these elements are fully configurable through a graphical user interface, no software development experience is needed to build a CTMS or a customized RDMS with the OAS from OpenCampus. The data cleaning process can already be implemented in the field verification and sanitization by checking that the inputted data has the right format and reasonable values with regard to the expected range values. For examples a field age can be allow only values ranging from 0 to 120 years and entered values higher 100 are highlighted in red font.

### Interoperability

There are taxonomies that allows to classify content with terms that are gathered within vocabularies. Using taxonomies the field content can be stored not as a text, but as a reference linked to predefined values that can be downloaded open source. For examples ICD-10 codes for a specific diagnosis. Furthermore, field content can be operationalized to avoid typos and misclassification (field: “sex” only “men”/”women” are applicable as a preset).

### Implementation in clinical research

A use case of OpenCampus will be presented in the following chapter, describing its implementation and customization in the Institute for Preventive Paediatrics at the Faculty of Sport- and Health Sciences of the Technical University of Munich.

**Fig. 2:**
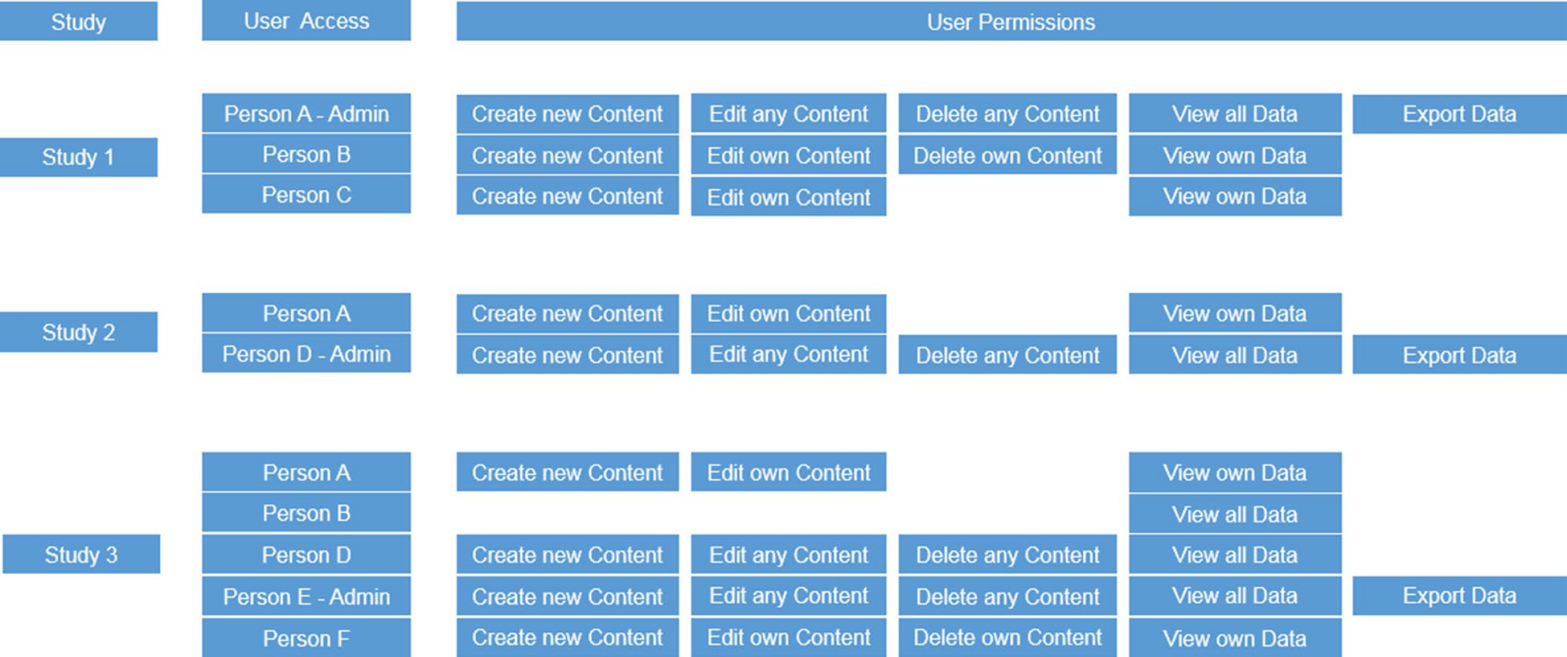
Structure of the accessibility and permission according to several studies

At our Institution, several research studies are managed by numerous researchers. Figure 2 presents the structure of the access and permission management map for the various studies and researchers. The administrator responsible for the study has full access to the data and can view, edit, delete and export data sets.

Due to the complex structure of the studies in terms of the different levels of researchers and contributors, the importance of providing custom access and permission rights to individuals is paramount. For example, we involved a student research assistant (Study 1, Person C) whose access was restricted to solely creating new data, editing own data but not to delete or export the data. He was also just permitted to view his own published data.

These various levels of access and permissions control can be customized for each individual researcher across the various studies and institutes.

### Multi-center

**Fig. 3:**
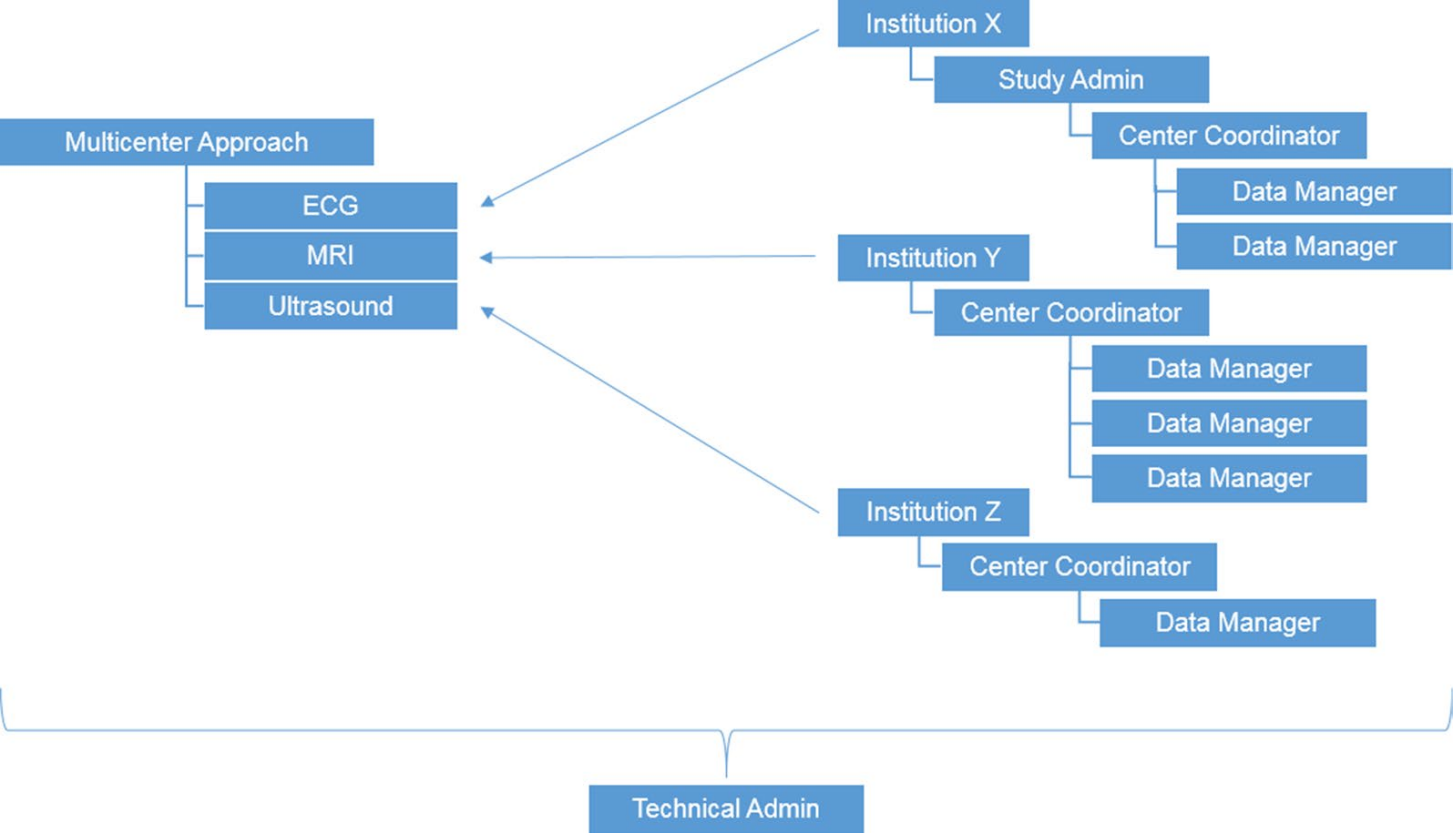
Multicenter approach

In a multi-center approach this concept works similar (Fig. 3). There is one single study administrator that assigns permissions to center coordinators. Center coordinators then independently distribute access and permissions to the data managers that are responsible for entering the data.

The granularity of the permission management concept allows each data manager to be able to access (within the group of accessible subjects of a center) data forms (nodes) like diagnostic data as ECG examination, Magnet resonance imaging (MRI) or ultrasound.

Data managers can now collect data in each study adding subjects and/or adding examinations. Using individual output “Views” they can display data they entered in various formats. Center coordinators can use output views to list and export data entered by any of his data managers. The study admin can use Views to list and export data, entered by any of the study centers within the respective study.

This concept ensures a convenient, location independent approach to collect study data, giving the peripheral units (study centers) full control to manage their own data pool and data base users. In addition, this feature offers all study centers the benefit of a supporting environment for patient care processes such as automated creation of clinical or statistical reports.

The multi center concept can be extended with various additional features such as node state levels or individual data processing guidelines that ensure that certain quality management actions are executed during data processing. This can be a mandatory review of data entered by a data manager: Each form filled in by a data manager is stored in ‘state 1’ after saving. Before the form is listed in the study data pool, it requires a review by a center coordinator who confirms the form by passing it into ‘state 2’.

### Meta-analysis

One core element of the generic data storage approach in the OpenCampus OAS concept allows to connect nodes to each other. This link between nodes is called ‘entity reference’. Using entity references the data from multiple studies can be linked (merged), allowing meta-analysis to be executed just by creating a new output view.

**Fig. 4:**
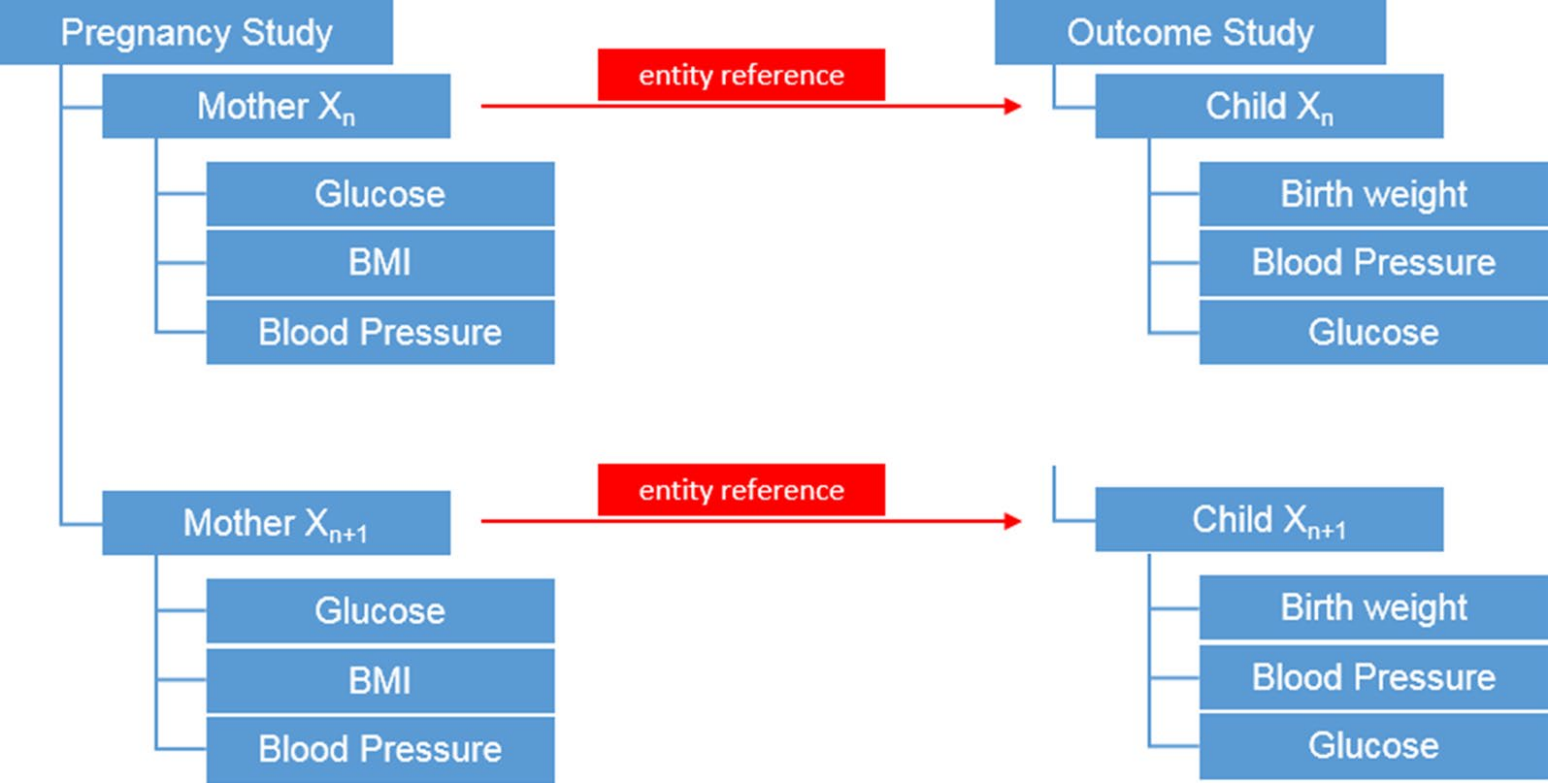
Meta-analysis

For example we conducted a “Pregnancy Study” where we examined women throughout their pregnancy, monitoring glucose, BMI and blood pressure (Fig. 4). Later we conducted an “Outcome Study” in which the childrens’ birth weight, blood pressure and glucose were investigated. We then connected the nodes of the children with the nodes of the mothers and created an entity reference between the two studies that allowed us to create an output ‘View’ that displayed characteristics from the children along with characteristics of their mothers. No data import was necessary at this point.

Furthermore, links between examinations or other data nodes are possible, e.g. to cross evaluate all examinations for one patient that were performed during multiple studies.

### Multi center data merge

**Fig. 5:**
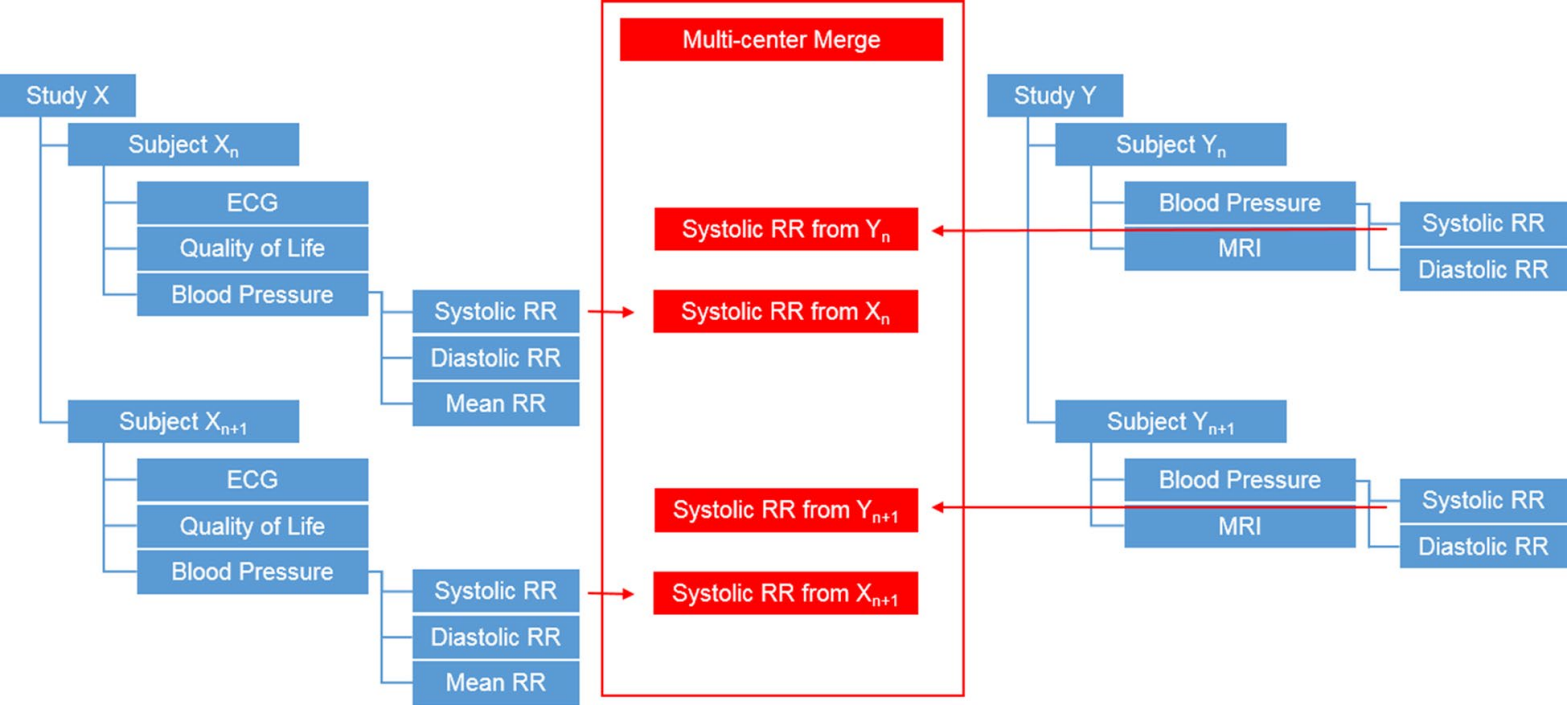
Multi center data merge

It is also possible to merge contents from completely independent studies. As seen in Fig. 5, in a study “X” blood pressure was measured as a part of the research. Another study “Y” also contained blood pressure, measured in a comparable method and procedure. Thus it is easily possible to merge the data from those two, or even more studies or to compare it against each other.

### Synergy between data collection and patient care operations

The RDMS provides condition based actions and outputs into formats such as email, PDF etc. which offers a seamless transition into patient care operations. At the same time, the system can provide us with medical reports, event planning, reminders and end user interaction. In clinical practice that allows examination data entered into OpenCampus, to be printed as a medical report and handed to the patient. Furthermore, a patient whose data were stored for medical purposes only can still give his consent online via OpenCampus, allowing the study center to use their data additionally for research purposes.

### Exporting and importing configurations

Using the export function, study sets (the entire RDMS or even complete CTMS) can be exported or cloned as a ‘configuration’ for further studies. This includes all Forms, Trees and Actions performed by the CTMS. With this method, even complex clinical trial sets can be shared across institutions, reducing the initial configuration effort for an institution and providing a way to share best practice models and transfer knowledge in a very compact and efficient manner.

### Data security

There are two major solutions. One possibility it the on-premise operation of the database with its physical location being within the hospital or patient care facility. The other possibility is a cloud based solution where signed patient consent is necessary. That complies also with the doctor-patient confidentiality. However the legal process is currently not fully reflected by the law.

## Limitations

Centrally stored data at a third party’s site do not receive legal protection in respect to any legal prosecution and consecutive seizures. In Germany this is regulated by an EVB-IT contract. In addition it should be considered that data protection regulations differ from country to country.

The flexibility of the OAS provides a high potential of individualization of field structure, data and content, which becomes a possible obstacle in multicenter studies when terminology is not uniform. It is therefore necessary to define distinctive entities for variable names, labels, types and units before implementing the system. For example variable name: height, label: “height in cm”, type: decimal, unit: cm, one digit right to the comma. Or for categorical variables, name: smoking, label: “smoking status”, type: list, unit: “none”, “former”, current”.

## Conclusions

With the OAS based OpenCampus system, a standard environment for study database design, user interface design and patient care support can be provided—ensuring that the entity relationship model of a study database is fully consistent and normalized, user interfaces are optimized for usability (access rights management, on screen data verification and calculation and web based data entry across all devices) and standard operating processes for patient care are supported or fully covered by the system. With a standard database technology and system framework used to operate within all clinical studies, meta-analysis of the existing data becomes an easy task. Not only within an institution but also as cross institutional meta-analysis in multi-center approaches. Due to the fully web based data collection approach, the OpenCampus technology allows location and device independent data entry.

This allows multi-center studies not only to be supported in a highly efficient manner but also to be managed and administrated in accordance with the legal and ethical guidelines of the scientific community.

The base technology for the OpenCampus RDMS being the open source development framework Drupal provides a robust and scalable platform technology. With a community of more than 1 million supporters it allows the integration of thousands of additional software modules available as free resources for Drupal.

In summary the described RDMS provides an open adoption software solution for research database management that allows non-technical individuals to build a consistent research database structure in line with modern and convenient user interfaces for data entry. The architecture of the technology provides unlimited scalability of interoperability between studies and study centers.

Drupal is a standard technology for many developers and researchers in a worldwide community, who extend, modify and contribute to this technology. This allows the integration of thousands of additional modules that can enhance the RDMS’s functionality.

## Abbreviations

CTMS: Clinical Trail Management System
OAS: open adoption software
RDMS: research data management system
